# Supplementary material for: Genome-wide association study reveals dynamic role of genetic variation in infant and early childhood growth
Source: Nat Commun. 2019 Oct 1;10:4448. doi: 10.1038/s41467-019-12308-0 (PMC6773698; doi:10.1038/s41467-019-12308-0)
Supplement: Supplementary file 4 — Description of Additional Supplementary Files [file 41467_2019_12308_MOESM4_ESM.pdf]

Description of Additional Supplementary Files

File Name: Supplementary Data 1
Description: Results for each of the five genome-wide significant markers in ADCY3 (rs13035244), FTO (rs9922708), LCORL (rs6842303), LEP (rs10487505) and LEPR (rs2767486) for the 12 time points analysed.

File Name: Supplementary Data 2
Description: Results from cell-stratified LD score regression at each of the 12 time points analysed in addition to tissue categories for GTEx v6p and Franke Lab annotations.
