# Supplementary material for: Genome-wide association study reveals dynamic role of genetic variation in infant and early childhood growth
Source: Nat Commun. 2019 Oct 1;10:4448. doi: 10.1038/s41467-019-12308-0 (PMC6773698; doi:10.1038/s41467-019-12308-0)
Supplement: Supplementary file 2 — Supplementary Information [file 41467_2019_12308_MOESM2_ESM.pdf]

Genome-wide association study reveals dynamic role of genetic variation in infant and early childhood growth

Helgeland et al.

Genome-wide association study reveals a highly dynamic role of common genetic variation in infant and early childhood growth

Øyvind Helgeland[1,2], Marc Vaudel[1], Petur B. Juliusson[3,4,5], Oddgeir Lingaas Holmen[6,7], Julius Juodakis[8], Jonas Bacelis[8,9], Bo Jacobsson[2,8], Haakon Lindekleiv[10], Kristian Hveem[6,11], Rolv Terje Lie[1,12], Gun Peggy Knudsen[2], Camilla Stoltenberg[12,13], Per Magnus[14,15], Jørn V. Sagen[1,16,3], Anders Molven[1,17,18], Stefan Johansson[1,19,20] and Pål R. Njølstad[1,4,20]**

[1] KG Jebsen Center for Diabetes Research, Department of Clinical Science, University of Bergen, NO-5020 Bergen, Norway.

[2] Department of Genetics and Bioinformatics, Health Data and Digitalization, Norwegian Institute of Public Health, NO-0473 Oslo, Norway.

[3] Department of Clinical Science, University of Bergen, NO-5020 Bergen, Norway.

[4] Department of Pediatrics and Adolescents, Haukeland University Hospital, NO-5021 Bergen, Norway.

[5] Department of Health Registries, Norwegian Institute of Public Health, NO-5020 Bergen, Norway.

[6] KG Jebsen Center for Genetic Epidemiology, Department of Public Health and Nursing, Faculty of Medicine and Health Sciences, Norwegian University of Science and Technology, NO-7491 Trondheim, Norway.

[7] HUNT Research Center, Department of Public Health and Nursing, Faculty of Medicine and Health Sciences, Norwegian University of Science and Technology, NO-7491 Trondheim, Norway.

[8] Department of Gynecology and Obstetrics, Sahlgrenska Academy, University of Gothenburg, SE-405 30 Gothenburg, Sweden.

[9] Department of Gynecology and Obstetrics, Sahlgrenska University Hospital, SE-413 45 Gothenburg, Sweden.

[10] University of Tromsø, NO-9019 Tromsø, Norway.

[11] HUNT Research Center, NO-7600 Levanger, Norway.

[12] Department of Global Public Health and Primary Care, University of Bergen, NO-5020 Bergen, Norway.

[13] Norwegian Institute of Public Health, NO-0473 Oslo, Norway.

[14] Centre for Fertility and Health, Norwegian Institute of Public Health, NO-0473 Oslo, Norway.

[15] Institute of Health and Society, Faculty of Medicine, University of Oslo, NO-0315 Oslo, Norway.

[16] Hormone Laboratory, Haukeland University Hospital, NO-5021 Bergen, Norway.

[17] Department of Clinical Medicine, University of Bergen, NO-5020 Bergen, Norway.

[18] Department of Pathology, Haukeland University Hospital, NO-5021 Bergen, Norway.

[19] Department of Medical Genetics, Haukeland University Hospital, NO-5021 Bergen, Norway.

[20] Joint senior authors: S. Johansson, P.R. Njølstad.

*** Address correspondence to: Professor Pål Rasmus Njølstad, M.D., Ph.D., K.G. Jebsen Center for Diabetes Research, Hospital for Children and Adolescents, Haukeland University Hospital, NO-5021 Bergen, Norway (pal.njolstad@uib.no); or to Professor Stefan Johansson, Ph.D., K.G. Jebsen Center for Diabetes Research, Department of Medical Genetics, Haukeland University Hospital, NO-5021 Bergen, Norway (stefan.johansson@uib.no)

Contents

Supplementary Figure 1	2
Supplementary Figure 2	3
Supplementary Figure 3	4
Supplementary Figure 4	5
Supplementary Figure 5	6
Supplementary Figure 6	7
Supplementary Figure 7	8
Supplementary Table 1	10
Supplementary Table 2	11

Supplementary Data 1 and 2 are available as spreadsheet in standalone files.

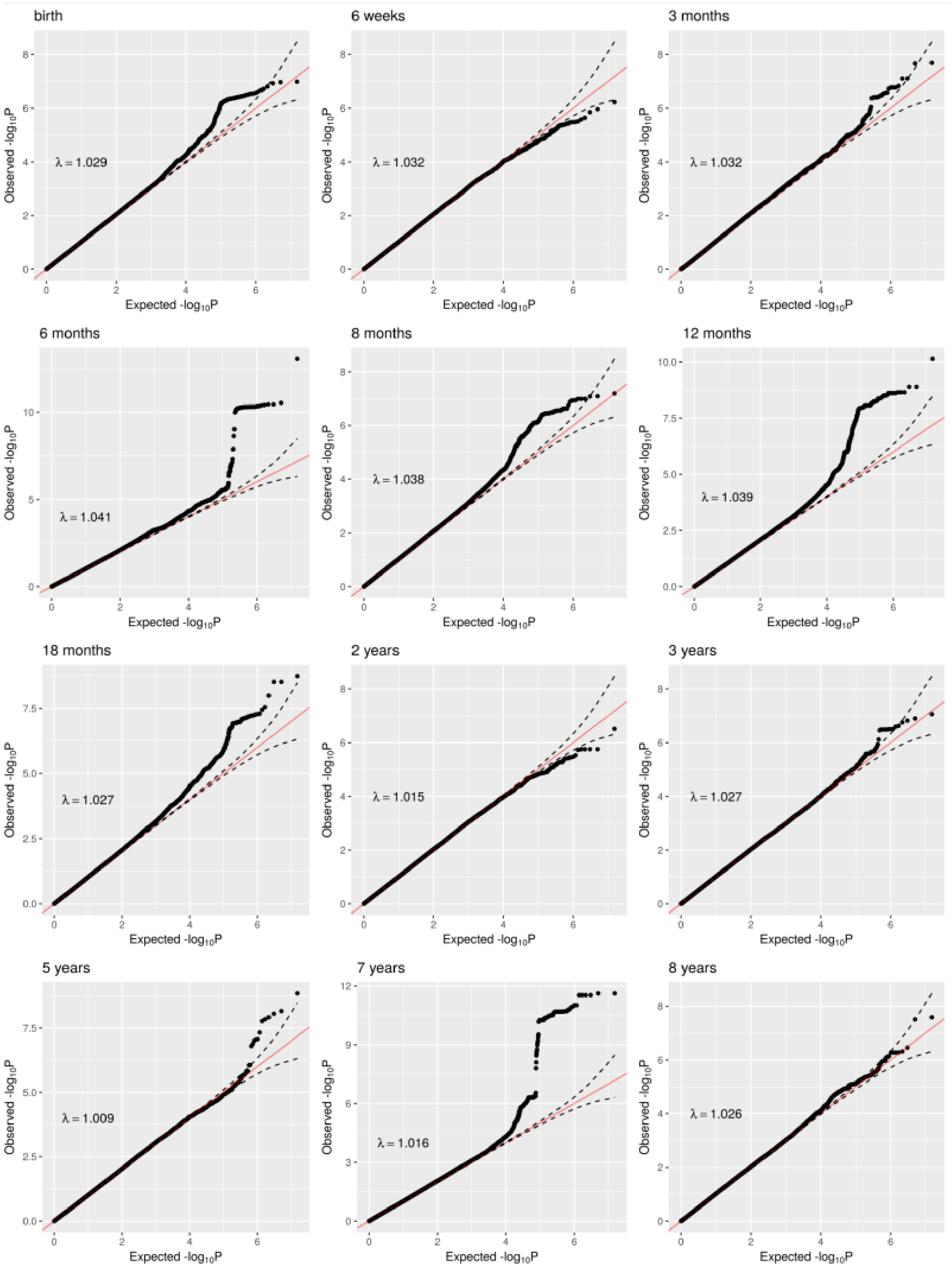

Supplementary Figure 1

QQ plots at all time points. QQ plots showing association results at birth, 6 weeks, 3, 6, 8 months, and 1, 1.5, 2, 3, 5, 7, and 8 years of age in the discovery sample. λ representing the inflation at median is indicated in the plot. The diagonal (y=x) is displayed in red and 95% confidence intervals as dashed black lines.

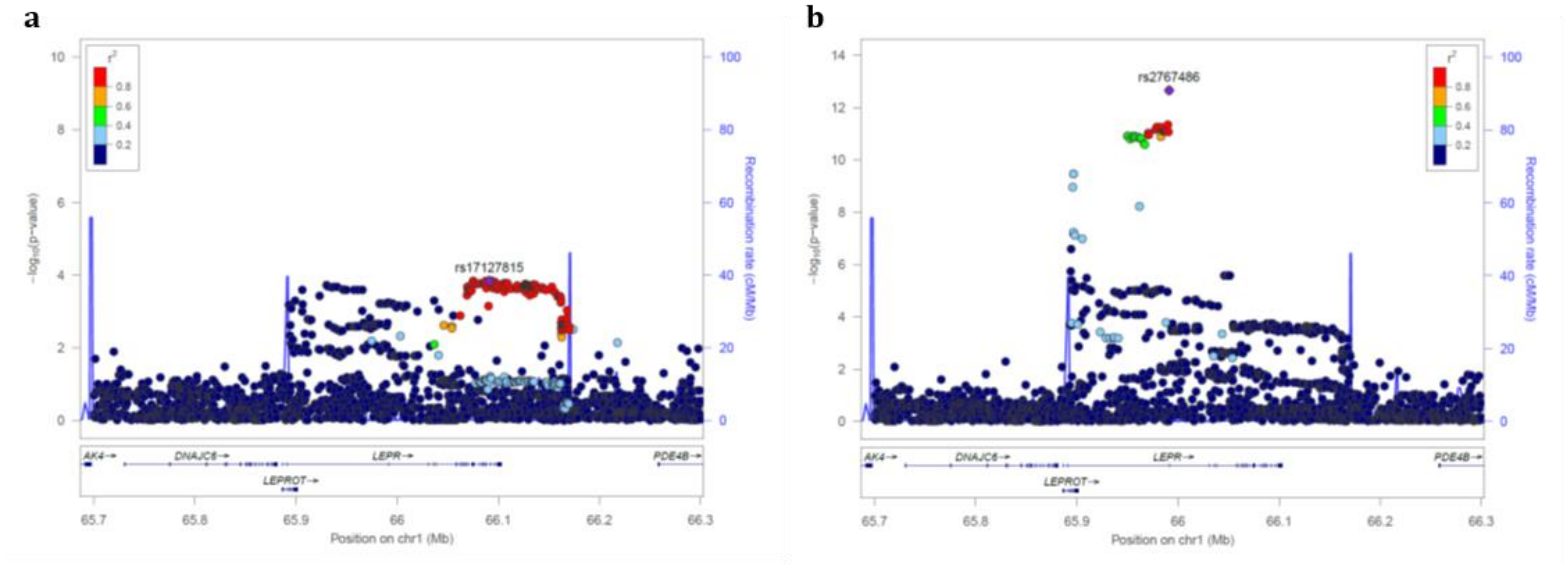

Supplementary Figure 2

Conditioning on variants associated with related traits. Regional association plot in the discovery sample in the *LEPR* locus at six months of age after conditioning on (a) rs2767485, the strongest pQTL for sOB-R-plasma levels in adults, and (b) rs11208659, associated with severe obesity. See main text for respective references.

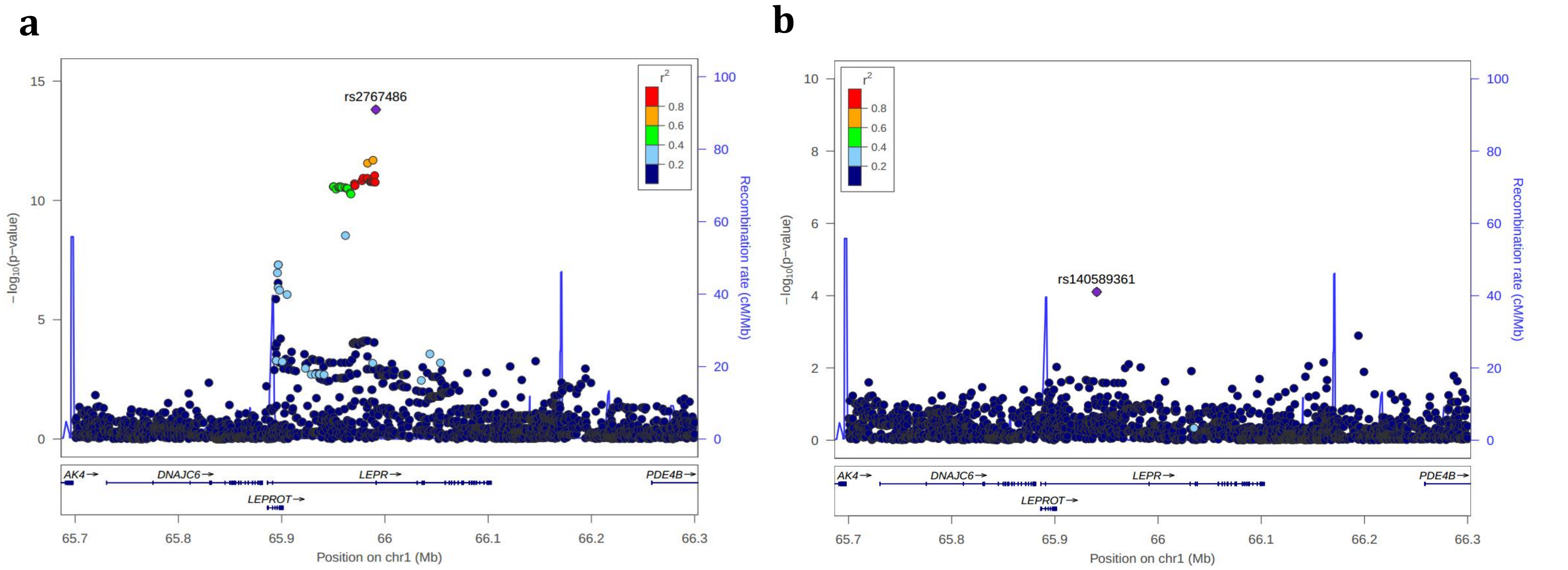

Supplementary Figure 3

Association in the LEPR locus. Regional association plot in the discovery sample after (a) conditioning on rs1805094 in the *LEPR* locus at 6 months, (b) conditioning on both rs2767486 and rs1805094 in the *LEPR* locus at 6 months

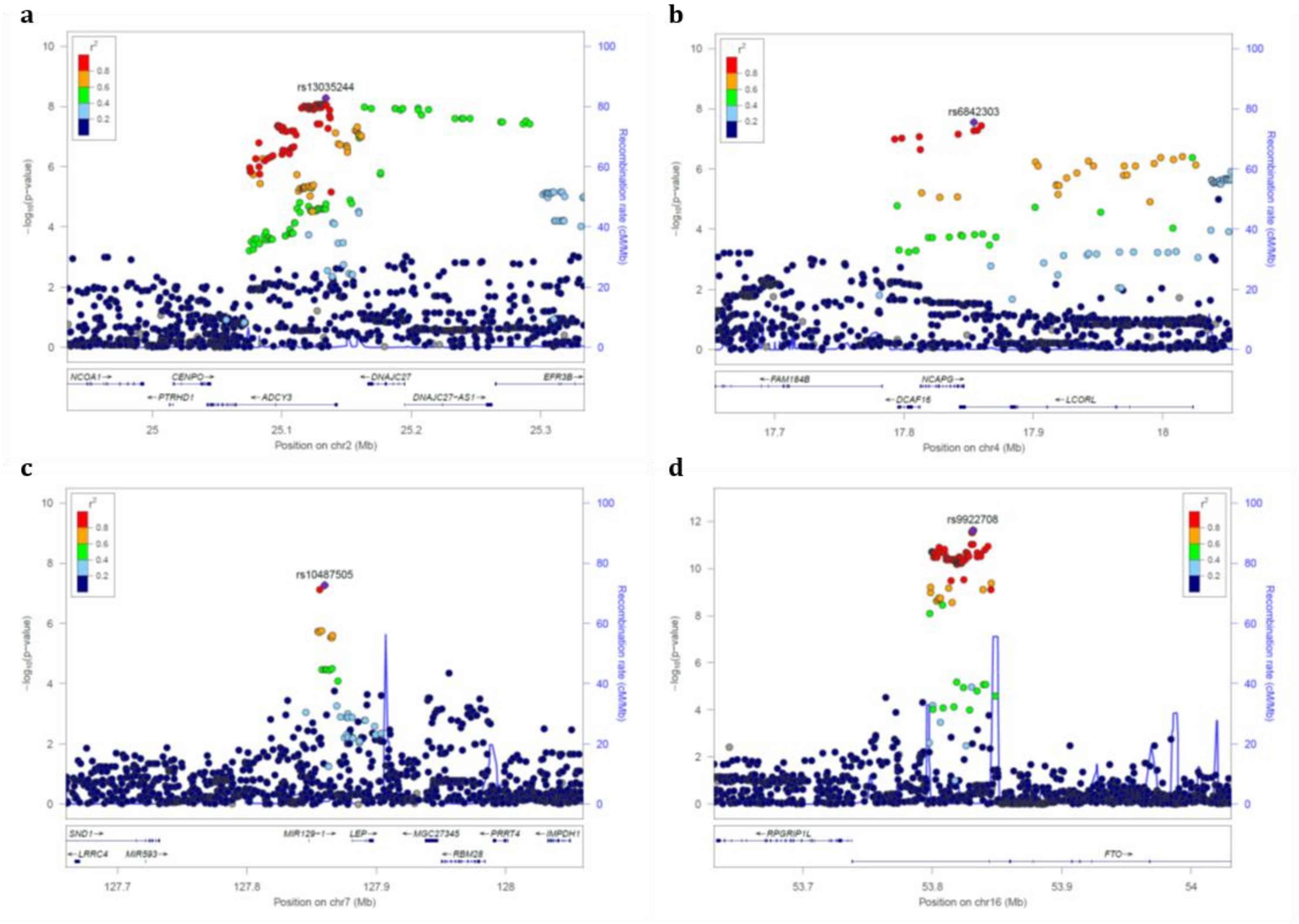

Supplementary Figure 4

Association in the *ADCY3*, *LCORL*, *LEP*, and *FTO* loci. Regional association plot in the discovery sample (a) rs13035244 in the *ADCY3* locus at 1 year, (b) rs6842303 in the *LCORL* locus at 1.5 years, (c) rs10487505 in the *LEP* locus at 1.5 years, and (d) rs9922708 in the *FTO* locus at 7 years.

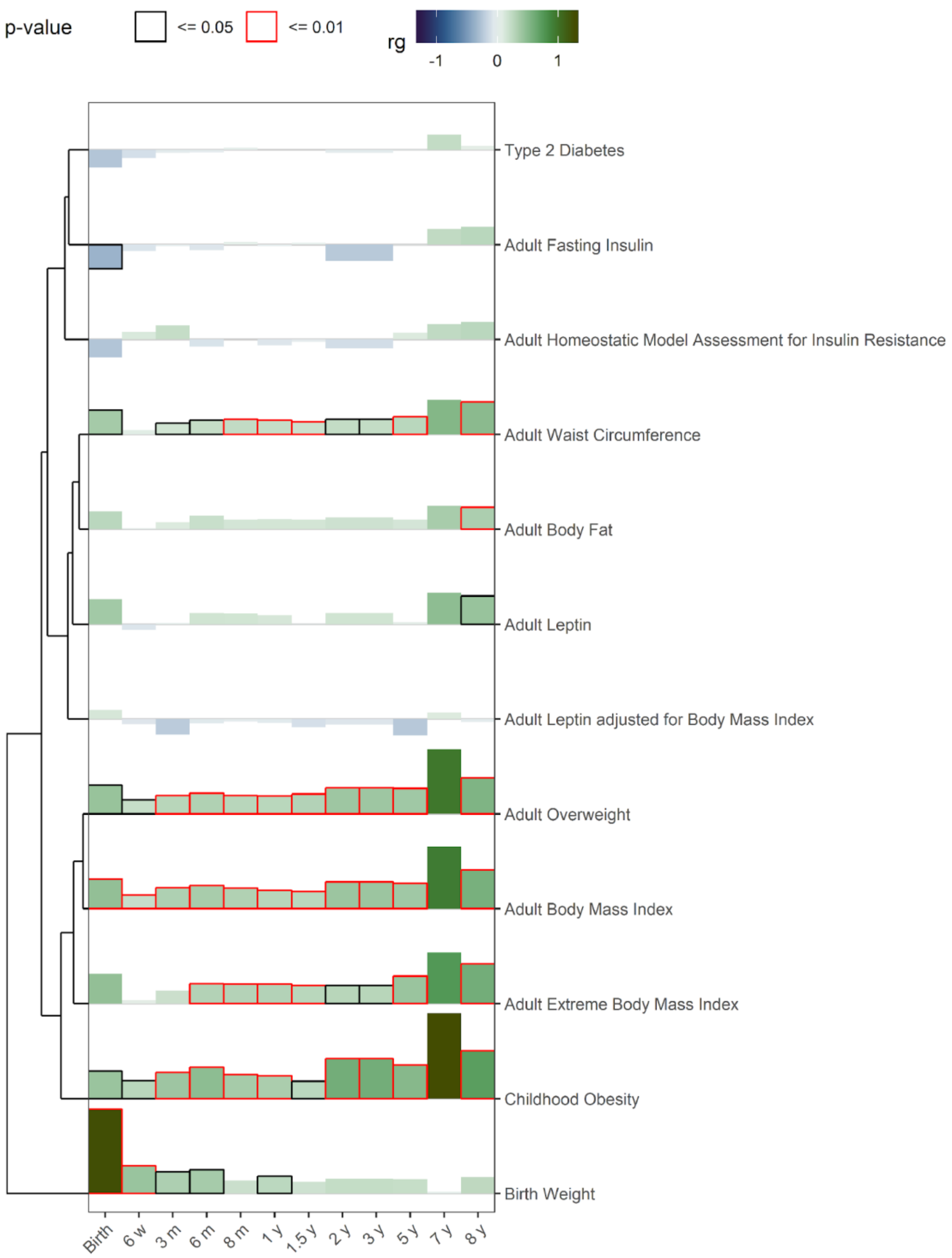

Supplementary Figure 5

LD-score regression coefficient (r_g) between BMI and related traits. The LD-score regression coefficient (r_g) between our BMI association results at each age and related traits is plotted at each time point as column of height and color representative of r_g. Data obtained from LD Hub (http://ldsc.broadinstitute.org/ldhub/) and calculated using LDSC v.1.0.0 (https://github.com/bulik/ldsc). Each column is outlined with a dark red and red square for p-values ≤0.05 and ≤0.01, respectively. The traits are ordered after hierarchical clustering as displayed with the dendrogram to the left: (i) T2D (Type 2 Diabetes), PMID 22885922; (ii) Fasting insulin main effect, PMID 22581228; (iii) homeostatic model assessment for insulin resistance, HOMA-IR (Homeostatic Model Assessment for Insulin Resistance), PMID 20081858; (iv) Waist Circumference, PMID 25673412; (v) Adult body fat, PMID 26833246; (vi) Leptin not adjusted for BMI, PMID 26833098; (vii) Leptin adjusted for BMI, PMID 26833098; (viii) Adult overweight, PMID 23563607; (ix) Adult BMI, PMID 20935630; (x) Extreme adult BMI, PMID 23563607; (xi) Childhood obesity, PMID 22484627; and (xii) Birth weight, PMID 23202124.

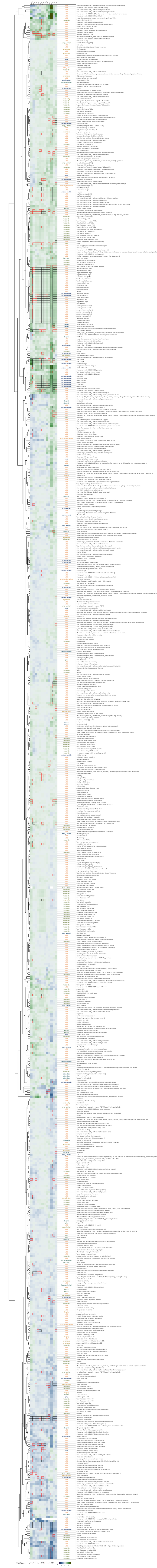

Significance
p > 0.05
p <= 0.01
p <= 0.05
rg
−1
0
1

Supplementary Figure 6

LD-score regression coefficient (rg) between BMI and other traits. The LD-score regression coefficient (rg) between our BMI association results at each age and other traits is plotted at each time point as heatmap of color representative of rg. Data obtained from LD Hub (https://ldsc.broadinstitute.org/ldhub/) and calculated using LDSC v.1.0.0 (https://github.com/bulik/ldsc). Each cell is outlined with a dark red and red square for P-values ≤0.05 and ≤0.01, respectively. The traits are ordered after hierarchical clustering as displayed with the dendrogram to the left.

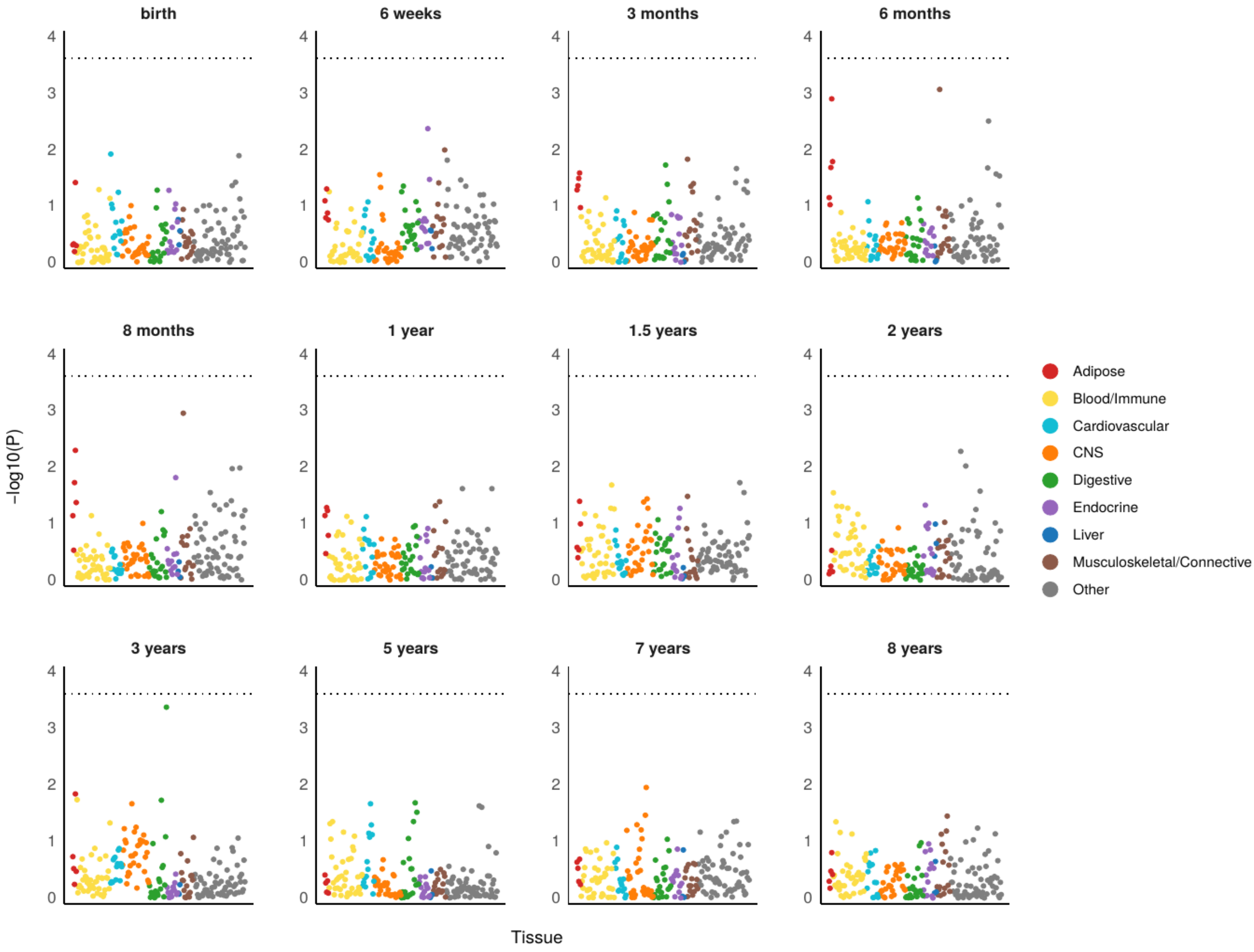

Supplementary Figure 7

Results of partitioned LD score regression analyses for selected traits. Each circle represents a tissue or cell type from the GTEx dataset or Franke Lab (total N = 37,427). The dotted line shows the -log10(P-value) threshold of 0.05 after Bonferroni correction for the 205 tissues present in the nine categories (numerical results and annotations available in Supplementary Table 4).

Age	Discovery			Replication			Merged		
	# Samples	Sex	BMI [Median (Q1 - Q3)]	# Samples	Sex	BMI [Median (Q1 - Q3)]	# Samples	Sex	BMI [Median (Q1 - Q3)]
Birth	9,286	F: 48.2%	F: 14.24 (13.45 - 15.12)<br>M: 14.28 (13.49 - 15.12)	5,235	F: 50.4%	F: 14.19 (13.34 - 14.99)<br>M: 14.24 (13.42 - 15.09)	14,521	F: 49.0%	F: 14.23 (13.40 - 15.08)<br>M: 14.26 (13.46 - 15.12)
6 w	6,450	F: 47.5%	F: 15.36 (14.51 - 16.20)<br>M: 15.87 (14.93 - 16.81)	3,444	F: 50.3%	F: 15.30 (14.38 - 16.22)<br>M: 15.83 (14.94 - 16.79)	9,894	F: 48.5%	F: 15.34 (14.48 - 16.21)<br>M: 15.86 (14.94 - 16.80)
3 m	7,970	F: 48.0%	F: 16.21 (15.33 - 17.20)<br>M: 16.76 (15.84 - 17.78)	4,453	F: 50.2%	F: 16.15 (15.26 - 17.20)<br>M: 16.77 (15.85 - 17.82)	12,424	F: 48.8%	F: 16.19 (15.30 - 17.20)<br>M: 16.77 (15.84 - 17.79)
6 m	8,017	F: 48.2%	F: 16.89 (16.00 - 17.93)<br>M: 17.35 (16.44 - 18.37)	4,516	F: 50.4%	F: 16.90 (15.99 - 17.91)<br>M: 17.31 (16.37 - 18.38)	12,531	F: 49.0%	F: 16.90 (16.00 - 17.93)<br>M: 17.34 (16.41 - 18.38)
8 m	7,197	F: 47.9%	F: 16.99 (16.06 - 17.95)<br>M: 17.44 (16.51 - 18.47)	3,966	F: 50.4%	F: 17.04 (16.09 – 18.00)<br>M: 17.48 (16.46 - 18.54)	11,161	F: 48.8%	F: 17.01 (16.06 - 17.96)<br>M: 17.45 (16.49 - 18.49)
1 y	6,970	F: 48.0%	F: 16.71 (15.85 - 17.62)<br>M: 17.14 (16.31 - 18.08)	3,839	F: 50.1%	F: 16.70 (15.87 - 17.66)<br>M: 17.17 (16.19 - 18.11)	10,808	F: 48.7%	F: 16.70 (15.85 - 17.64)<br>M: 17.15 (16.28 - 18.09)
1.5 y	6,800	F: 47.9%	F: 16.47 (15.65 - 17.35)<br>M: 16.94 (16.05 - 17.85)	3,780	F: 50.0%	F: 16.53 (15.68 - 17.51)<br>M: 16.93 (16.03 - 17.85)	10,578	F: 48.6%	F: 16.50 (15.66 - 17.42)<br>M: 16.93 (16.04 - 17.85)
2 y	4,631	F: 48.6%	F: 16.31 (15.43 - 17.23)<br>M: 16.54 (15.67 - 17.53)	2,502	F: 49.4%	F: 16.33 (15.35 - 17.31)<br>M: 16.66 (15.70 - 17.55)	7,131	F: 48.9%	F: 16.32 (15.40 - 17.27)<br>M: 16.57 (15.68 - 17.53)
3 y	5,069	F: 47.5%	F: 15.95 (15.09 - 16.98)<br>M: 16.14 (15.20 - 17.13)	2,834	F: 49.9%	F: 16.00 (15.00 - 16.98)<br>M: 16.17 (15.28 - 17.22)	7,905	F: 48.3%	F: 15.96 (15.05 - 16.98)<br>M: 16.15 (15.25 - 17.16)
5 y	4,245	F: 47.7%	F: 15.54 (14.58 - 16.53)<br>M: 15.52 (14.65 - 16.52)	2,208	F: 51.0%	F: 15.39 (14.46 - 16.35)<br>M: 15.43 (14.57 - 16.35)	6,454	F: 48.8%	F: 15.43 (14.51 - 16.47)<br>M: 15.49 (14.61 - 16.47)
7 y	4,629	F: 47.4%	F: 15.61 (14.60 - 16.83)<br>M: 15.59 (14.65 - 16.68)	2,638	F: 50.3%	F: 15.45 (14.46 - 16.72)<br>M: 15.50 (14.59 - 16.58)	7,268	F: 48.5%	F: 15.53 (14.57 - 16.80)<br>M: 15.56 (14.63 - 16.65)
8 y	3,862	F: 47.9%	F: 15.98 (14.85 - 17.36)<br>M: 15.92 (14.88 - 17.16)	2,087	F: 50.3%	F: 15.98 (14.80 - 17.34)<br>M: 15.87 (14.86 - 17.01)	5,949	F: 48.7%	F: 15.98 (14.81 - 17.36)<br>M: 15.91 (14.88 - 17.12)

Supplementary Table 1

Summary statistics on the phenotypes used for association. At each time point, the number of samples, proportion of females (F), and BMI summary statistics are listed for females (F) and males (M). The BMI summary statistics provided are median and Q1 and Q3 the first and third quartiles, respectively. Summary statistics are provided for (i) the discovery cohort, (ii) the replication cohort, and (iii) both cohorts merged. Note that in the latter case, outlier detection is conducted in the entire cohort, yielding minor differences between the number of samples in the merged cohort compared to the sum of samples in discovery and replication

	Discovery			Replication		
Age	Total samples used for analysis (including imputed values)	Samples with available height and weight measurements	Difference in Percent	Total samples used for analysis (including imputed values)	Samples with available height and weight measurements	Difference in Percent
Birth	9286	9286	0%	5235	5235	0%
6 w	6450	6450	0%	3444	3444	0%
3 m	7970	7955	0%	4454	4447	0%
6 m	8017	7791	3%	4516	4452	1%
8 m	7197	6489	10%	3966	3561	10%
1 y	6970	6488	7%	3839	3578	7%
1.5 y	6800	6552	4%	3780	3595	5%
2 y	4631	3487	25%	2501	1808	28%
3 y	5069	4699	7%	2833	2621	7%
5 y	4245	4258	0%	2209	2216	0%
7 y	4629	4670	-1%	2638	2671	-1%
8 y	3862	3862	0%	2087	2087	0%

Supplementary Table 2

Summary statistics on the number of phenotypes available at each time point before and after extreme outlier removal and imputation of sporadic missing values in Discovery and Replication cohorts. At each time point, the number of samples (i) total used for analysis, including imputed height and/or weight (ii) with complete information of height and weight available for BMI calculation, and (iii) the difference in percent. The extreme outlier removal and imputation strategy is described in detail in the materials and methods section and involved imputation only of sporadic missing values preceded and followed by at least two non-imputed values. A positive difference indicates a gain in number of samples, the negative difference, i.e. a loss of samples, observed at 7 years of age is the result of outlier removal at an age where no imputation is possible according to our imputation criteria.
